# Advances in MUC1-Mediated Breast Cancer Immunotherapy

**DOI:** 10.3390/biom12070952

**Published:** 2022-07-06

**Authors:** Zhifeng Li, Dazhuang Yang, Ting Guo, Mei Lin

**Affiliations:** 1 Medical School of Nantong University, Nantong 226019, China; 2013510032@stmail.ntu.edu.cn (Z.L.); yang_dzhuang@163.com (D.Y.); 2 Research Center of Clinical Medicine, Jiangsu Taizhou People’s Hospital (Affiliated Hospital 5 of Nantong University), Taizhou 225300, China; wangjunguoting2008@163.com

**Keywords:** mucin, MUC1, breast cancer, target, immunotherapy

## Abstract

Breast cancer (BRCA) is the leading cause of death from malignant tumors among women. Fortunately, however, immunotherapy has recently become a prospective BRCA treatment with encouraging achievements and mild safety profiles. Since the overexpression and aberrant glycosylation of MUC1 (human mucin) are closely associated with BRCA, it has become an ideal target for BRCA immunotherapies. In this review, the structure and function of MUC1 are briefly introduced, and the main research achievements in different kinds of MUC1-mediated BRCA immunotherapy are highlighted, from the laboratory to the clinic. Afterward, the future directions of MUC1-mediated BRCA immunotherapy are predicted, addressing, for example, urgent issues in regard to how efficient immunotherapeutic strategies can be generated.

## 1. Introduction

Breast cancer (BRCA) is a malignant epithelial tumor of ductal or lobular origin. According to IARC statistics, BRCA ranked first in terms of its morbidity worldwide and second with regard to factors inducing cancer-associated mortality in 2020. There were over 2.16 million new cases and 618,000 deaths from BRCA worldwide in 2020, and these two numbers are expected to reach 3 million and 1 million, respectively, in 2040 [1]. Excluding the gender factor, the incidence of BRCA among females far exceeds any other tumor. Ductal carcinoma in situ (DCIS) and non-invasive BRCA can be cured by surgery. Contrastively, invasive BRCA requires comprehensive treatment based on surgery, in order to achieve long-term survival. Invasive BRCA can be classified into different molecular subgroups (such as luminal A/B, triple-negative, and HER2-positive), and they show great differences in treatments (such as surgery, radiotherapy, chemotherapy, molecular targeted therapy, and endocrine therapy) and prognostic outcomes [2]. Despite significant advances in these therapies, BRCA is still a major factor causing cancer-associated mortality in females. With the rise of tumor immunotherapy over the last three decades, many investigators have sought an immunotherapeutic approach to treating BRCA. BRCA immunotherapy includes diverse therapeutic modalities such as vaccinations, monoclonal antibodies (mAbs), adoptive T-cell transfer immunotherapy, and immune checkpoint inhibitors (ICIs). Some immunotherapeutic approaches, such as that based on immune checkpoint inhibitor PD1/PD-L1, are becoming an important part of BRCA therapy as they show favorable therapeutic effects and a high level of safety. Nonetheless, due to the lack of therapeutic targets, current immunotherapies are only effective in certain patient populations, so it is necessary to develop novel immunotherapy targets and methods in order to benefit more BRCA patients. In the process of exploring BRCA immunotherapy targets, mucin 1(MUC1), a hyperglycosylated, high molecular weight protein expressed by the MUC1 gene, continues to arouse interest. Within the last three decades, MUC1 has been recognized as the candidate anticancer therapeutic target due to its upregulated expression, abnormal glycosylation, and polarization loss within numerous adenocarcinomas. In addition, MUC1 expression can be detected within certain hematopoietic malignancies, including myeloma and acute myeloid leukemia (AML). Several clinical studies are being conducted and immune responses are being documented. The efficient clinical benefits in general applications have not been approved [3]; however, MUC1 is still attractive as an immunotherapeutic target for BRCA, which is evidenced by the increasing number of MUC1-based BRCA immunotherapy research articles initiated in 2016. Herein, the exploratory research published on pubmed.gov (accessed on 11 May 2022) and the clinical trials registered on clinicaltrials.gov (accessed on 11 May 2022) are presented and analyzed, and the attempts made to date are summarized, so as to bring MUC1 into the world of BRCA immunotherapy.

## 2. Overview of MUC1

**Figure 1:**
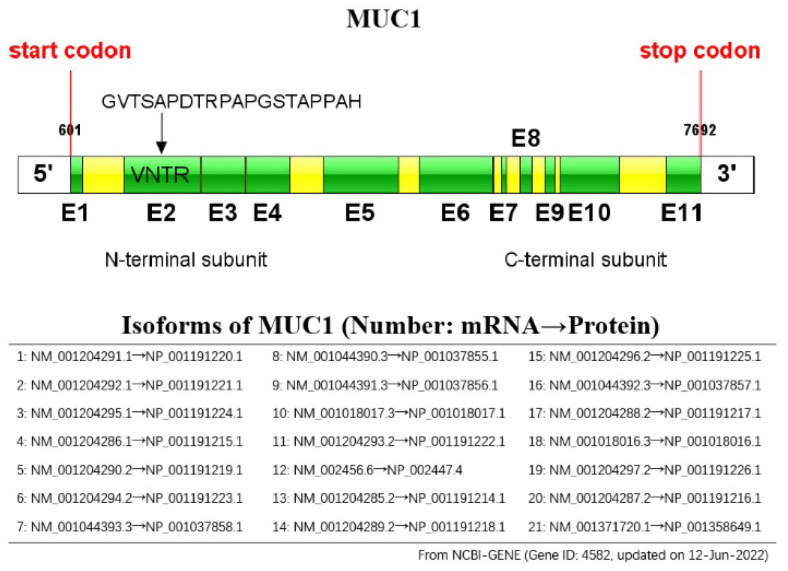
Schematic representation of the MUC1 gene: the MUC1 gene consists of 11 exons (E1 to E11, indicated by green colored boxes), and has 21 isoforms.

**Figure 2:**
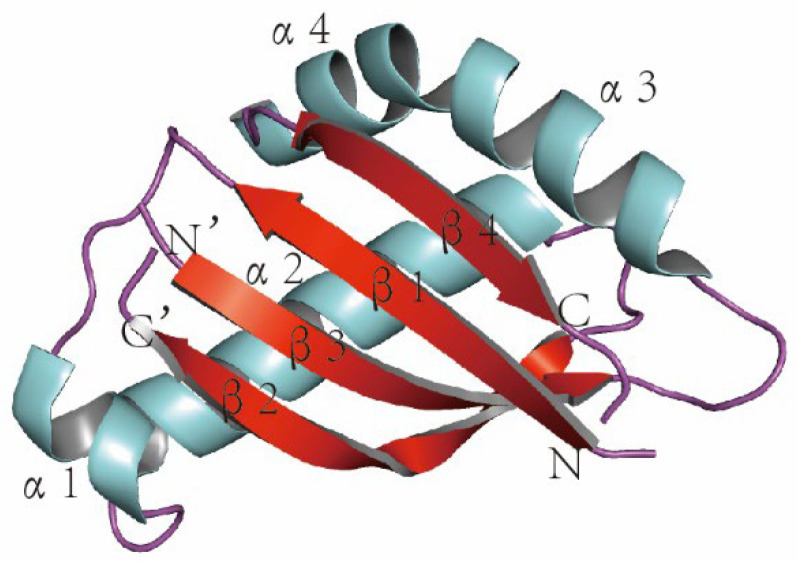
The 3D structure of MUC1 (colored by secondary structure), α: alpha spiral, β: beta fold, N: NH_2_, and C: COOH. The 3D coordinate of the MUC1 structure (PDB ID: 2ACM) was retrieved from the Protein Data Bank (http:/www.rcsb.org/pdb/home.do) (accessed on 2 March 2022). The graphic was created by PyMOL (version 0.99; DeLano Scientific, San Carlos, CA, USA).

The human MUC1 gene is located on chromosome 1q22 and contains 11 exons, from which, 21 MUC1 isoforms are derived due to the many variable numbers of tandem repeats (VNTRs) contained in these exons (Figure 1). It is responsible for encoding one single-polypeptide chain, which can be cleaved through the auto-proteolysis of the sea-urchin sperm protein, enterokinase, and agrin (SEA) domain for generating two peptide fragments, the short C-terminal (MUC1-C) and long N-terminal (MUC1-N) subunits [4,5]. The MUC1 protein was the initial mucin for structural characterization and the initial membrane mucin for identification [6,7,8,9] (Figure 2). Moreover, the MUC1 protein is a heterodimer consisting of two subunits and is cleaved during translation [10,11]. MUC1-N involves regions of VNTRs that may be subject to glycosylation on serine/threonine residues. Every VNTR contains 20 amino acids (AAs) (GVTSAPDTRPAPGSTAPPAH), whereas MUC1-C belongs to the heterodimeric transmembrane member that anchors MUC1-N onto the cell surface [12]. Thereafter, MUC1-N can be generated on cell surfaces while MUC1-C plays the role of the candidate receptor for intracellular signaling [13].

MUC1 gene expression is regulated at the transcriptional level through the inter-action between cis-acting elements in the MUC1 promoter and transcription factors in the cell. The MUC1 promoter contains two Sp1 binding sites, one SpA binding site, and one E-box (E-MUC1). The interaction between Sp1 and its binding site promotes MUC1 gene expression, while SpA downregulates its expression. The ratio of Sp1 to SpA may be an important factor in determining the level of MUC1 gene expression. The high level of MUC1 expression in tumor cells may be associated with dysregulation between the two. The Sp1 locus and E-box (E-MUC1) may be associated with the tissue-specific expression of MUC1.

MUC1 plays the role of a barrier within normal tissues for cell protection via the extracellular domain [14]. Membrane-bound MUC1 is the physical barrier, which exerts its effect via the extracellular SEA domain. To be specific, it modulates cell adhesion and shedding in the process of metastasis, prevents epithelial cell apical membrane rupture, immune invasion, and adverse environmental conditions, induces stimulus resistance, suppresses immune reactions by means of receptor shielding, and acts as the decoy receptor for pathogenic microorganisms [15]. It is also related to hydration on the cell surface, lubrication, and resistance to degrading enzymes [16,17,18,19].

**Figure 3:**
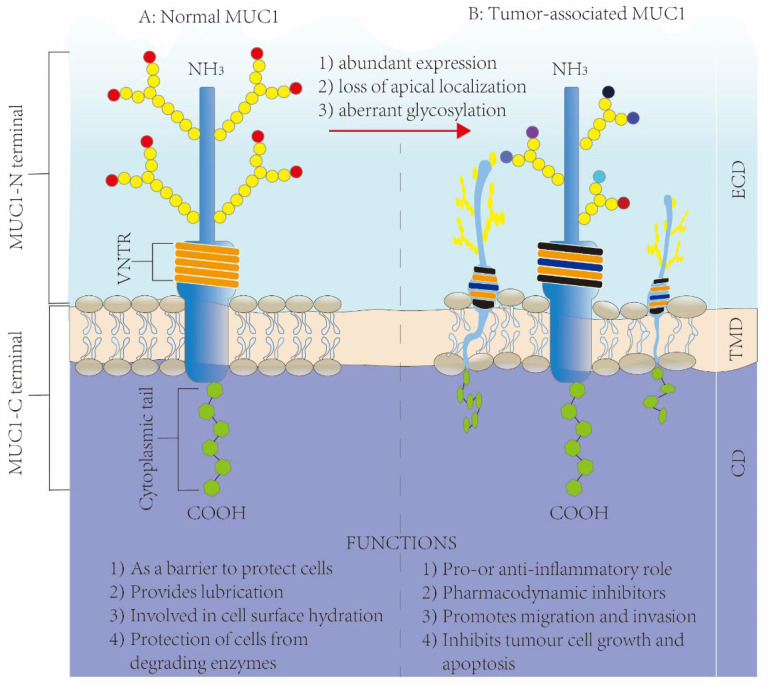
Structure and functions of MUC1 in normal tissues and diseased tissues: (**A**) the structure of MUC1 in normal tissues; (**B**) the structure of MUC1 in tumor tissues.

MUC1 has substantially altered roles within cancer tissues, which is closely related to tumor development and treatment. The overexpressed MUC1 level within BRCA can be ascribed partially to transcription disturbance and genetic variations. Gene MUC1 is on chromosome 1q22, and this region is usually different within BRCA cells [20]. It is aberrantly overexpressed in over 90% of BRCA samples which is markedly related to the upregulated MUC1 protein and mRNA expression [21,22,23]. Apart from that, according to prior works and research on human mucin MUC1, there are specific characteristics of tumor-related MUC1, including (1) a high abundance [24], (2) apical localization loss as well as alterations to a peripheral one [25,26], and (3) abnormal glycosylation that induces tumor-related antigen epitope formation [27,28,29] (Figure 3). These tumor-associated changes further affect a variety of tumor progression pathways [5]; one pathway is its anti- and pro-inflammatory activities within diverse infection-caused tumors via the role of an immunomodulatory switch [14,30] and another is pharmacodynamic inhibitors. On the other hand, MUC1 also results in chemoresistance [31] and radio-resistance [32] during cancer therapy; a third pathway is MUC1-promoting tumor invasion and migration. MUC1 can contribute to different tumor invasions and migrations by regulating tumor cell invasion and metastasis-related factors [33,34]. Other than that, MUC1 expression promotes tumor blood vessel formation and partially enhances tumor invasion and metastasis. In BRCA, the expression of MUC1 is closely related to that of vascular endothelial growth factor (VEGF), and MUC1 expression has been found to enhance blood vessel formation in BRCA patients both in vitro and in vivo [35,36]. A fourth pathway is MUC1’s suppression of tumor cell proliferation and apoptosis, which is related to regulating diverse tumor cell proliferation/apoptosis pathways. Typically, in BRCA, epithelial cell polarity loss and transformation induce an overexpressed MUC1 level, and the overexpressed MUC1-C, but not MUC1-N, can then promote cells to resist stress-induced apoptosis while enhancing transformation [37,38]. MUC1 silencing has been suggested to suppress cell growth, cause apoptosis, and promote cell-cell aggregation [39].

The MUC1 protein is related to the interactions between cells or between cells and the extracellular matrix (ECM), which, thus, possibly promotes tumor cell metastasis at the primary site [40]. MUC1 participates in carcinogenesis and can promote tumor occurrence and development [41]. Moreover, mucin-like molecules are related to a passive effect on regulating cell migration and adhesion, as well as an active effect on signal transduction [42]. Endothelial mucin-like factors such as GlyCAM1, CD34, MadCAM1, and PSGL1 [43] can interact with corresponding ligands and are thus associated with signal transmission to the nuclei and lymphocyte trafficking. In addition, MUC1 also has numerous additional effects on cancer development and epithelial morphogenesis, because it is extensively expressed within the secretory epithelium from mid-gestation through all of adulthood; in particular, MUC1 overexpression can be detected in tumors as well as metastases [44]. In addition, MUC1 has been suggested to offer steric hindrance via its great extracellular glycosylated domain [45], cytoskeletal network remodeling [46], and the reduction in signal transduction events through the actions of catenin, integrins, and cadherins [47]. Beyond that, MUC1’s cytoplasmic tail can be subject to phosphorylation within BRCA cells, thus supporting MUC1’s transmembrane signaling activity [48]. So far, at least five isoforms of MUC1 have been identified, namely, MUC1/REP, MUC1/SEC, MUC1/X, MUC1/Y, and MUC1/Z. Notably, MUC1/Y is identified as MUC1’s special differentially splicing product and is specifically expressed in tumors. The MUC1/SEC serves as MUC1/Y’s ligand, and it functions in initiating signaling events while changing cancer cell morphologies [49]. Based on the above results, MUC1 is promising as the perfect antitumor therapeutic target. Its activities possibly affect tumor development, thereby offering data supporting its role as an immunotherapeutic target.

## 3. MUC1-Mediated BRCA Immunotherapy

**Figure 4:**
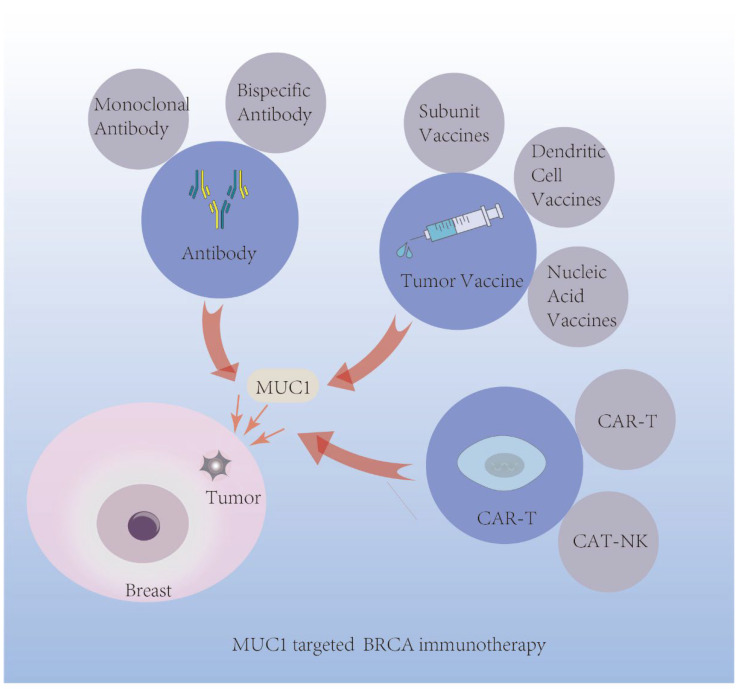
Application of MUC1 mediated immunotherapy in BRCA.

In terms of BRCA immunotherapy, MUC1 research has followed three main lines [3]. MUC1’s glycopeptide epitope is one line, which has long been identified to induce cellular/humoral adaptive immunity [50]. Tumor cell-expressed MUC1 is another line, and it has a certain effect on immunocyte function and phenotypes within the tumor microenvironment (TME). A third line is MUC1-C, the small membrane-spanning protein. Based on these lines, MUC1 is designed in antibody-based, vaccine-based, and CAR-T-cell therapies (Figure 4).

### 3.1. MUC1 Antibody-Based Therapeutics

Antibody-based immunotherapy has been employed for cancer treatment within the past few decades and is currently one of the most effective strategies for treating hematological cancers and solid tumors [51]. Antibody-based therapeutics can also target tumors in a highly specific and efficient manner. As mentioned above, MUC1 represents a critical tumor marker that is highly expressed within BRCAs, so it has become a possible antibody-based therapeutic target. Furthermore, the preclinical and clinical research of related MUC1 antibody-based BRCA immunotherapy focuses on three areas: monoclonal antibodies, nanobodies, and bispecific antibodies against MUC1.

#### 3.1.1. Monoclonal Antibodies (mAbs)

mAbs, synthesized immune system proteins, are synthesized and engineered for binding to corresponding tumor cell epitopes [52,53]. They kill tumor cells via multiple mechanisms which include macrophage phagocytosis, inducing apoptosis, suppressing target activity, introducing immune system components, and delivering radiotherapy/chemotherapy agents in targeted treatments [54,55]. Tumor antibody-based therapies are dependent on upregulation, mutation, or restricted expression of tumor cell surface antigens [56]. Furthermore, the major characteristics making MUC1 an appropriate antibody-based therapeutic target are a significantly increased MUC1 expression within epithelial tumors, an apical-basal polarity loss of tumor cells, and MUC1 expression on a cell surface. Consequently, it can access tumor-specific truncated carbohydrate antigens, such as TF and Tn, within MUC1’s VNTR region, as well as antibodies, and can reduce glycan site occupancy [3]. In this case, after considering challenges such as the suitable design of antibodies, therapeutic agents based on the MUC1 antibody could be effective and safe anti-tumor therapeutic approaches. Therefore, antibodies are being prepared based on diverse MUC1 domains, including one portion in clinical trials and another in experimental or preclinical research. The present work discusses several anti-MUC1 antibody-based therapies that have been frequently used in experimental, preclinical, and clinical studies within the past few decades (Table 1).

- huHMFG1 (also named AS1402, formerly R1550)

The human milk fat globule 1 (HMFG1) is a humanized IgG1k monoclonal antibody that can recognize the PDTR epitope in MUC1 ECD’s VNTR region. huHMFG1 is a fully-humanized format of huMFG1, produced through the transfer of mouse HMFG1’s complementarity determining regions (CDRs) [87]. It can potently induce antibody-dependent cellular cytotoxicity (ADCC), in particular, for cancer cells that express MUC1. huHMFG1 was first developed as a monotherapy, and its tolerability, safety, and pharmacokinetic (PK) properties were investigated in a Phase I trial (NCT00096057). Twenty-six MUC1-expressing BRCA women with metastases or locally advanced lesions who showed disease progression following taxane- and anthracyclines-based treatments were treated with huHMFG1. The results showed that repeated intravenous dosing of huHMFG1 was well tolerated and that effective therapeutic concentrations were maintained in vivo for a long period of time. However, no clinical therapeutic effects have been reported [57]. In a Phase II clinical trial (NCT00770354), the outcomes of patients suffering from BRCA treated with letrozole and huHMFG1 were compared with those treated only with letrozole. Due to the possibly high disease progression and poor response rates, the combination therapy was terminated early, and no safety concerns were reported [58].

- TAB004

TAB004, one of the humanized IgG1 mAbs, can recognize hypoglycosylated TA-MUC1’s STAPPVHNV sequence in a TR sequence. It can be used to assess circulating tMUC1 levels and aid the earlier diagnosis of BRCA, regardless of breast tissue density [59]. Mesoporous silica nanoparticles coated with mTAB004 (murine TAB004), fluorophore, and indocyanine green (ICG) were also successfully adopted for the early detection of BRCA in an animal experiment [60,61]. In another study, humanized TAB004 conjugated with 111In and 225Ac-DOTA exhibited superb tumor-targeting ability and achieved complete remission among TNBC cases [62]. Recently, a Phase I clinical trial (NCT04137900) evaluated the immunogenicity, PK profile, and anticancer effect of TAB004 in patients who had advanced solid tumors.

- BrE-3

BrE-3 is a type of IgG1, whose kappa murine monoclonal antibody binds to TRP of an AA sequence in VNTRs, and it contributes few carbohydrates to BRCA’s MUC1 epitope and binds to several healthy mammary epithelial cells [64]. Its several conjugated forms, including 111In-Methyl Benzyl DTPA BrE-3 [65] and 111In- and 90Y-labelled BrE-3 [63], are utilized to treat BRCA metastases. Nonetheless, humans have developed an anti-mAb response to mouse BrE-3 mAb; as a result, the humanized BrE-3 (huBrE-3) has been developed [66]. 111In huBrE-3 has attained superb cancer localization (76%), a half-time of 114.2 h during blood clearance, and undetectable anti-mAb response among BRCA cases, as suggested in a Phase I trial (NCT00007891) [67].

- DS6

As an IgG1 mouse antibody, DS6 can recognize MUC1’s CA6 sialoglycotope which shows overexpression in various solid tumor types, such as BRCA, ovarian cancer (OC), lung cancer (LC), pancreatic cancer, and cervical cancer (CC) [88]. CA6-expressing MUC-1 has the characteristics of mucin-type O-linked glycans that contain the β1,4-galactosylated terminal and the α2,3-sialylated terminal, and they can specify the binding of DS6 to the tandem repeat (TP) domain of MUC-1 according to the above three glycan structures. SAR566658, which is an ADC agent and is also the maytansinoid (DM4)-conjugated humanized DS6 (HuDS6), was utilized in a Phase I clinical trial (NCT01156870) on 114 solid tumor cases at diverse doses, which was safe and potent in 35–60% of cases. A Phase II clinical trial (NCT02984683) was further conducted to assess SAR566658 among CA6-expressing metastatic TNBC cases, but it was prematurely terminated because only low clinical benefits were obtained and the mild ophthalmological event rate was high [68].

- SM3

SM3, an IgG1λ mouse antibody, is reactive to the MUC1 VNTR’s non-glycosylated PDTR motif. It can interact with the protein core in antigens’ polymorphic epithelial mucin (PEM) within mammary ducts. Other than that, as suggested by an immunohistochemical (IHC) work concerning BRCA tissues, SM3 had a strong reaction with most primary BRCAs (91%), but it had no or little reaction with many healthy tissues and with lactating or resting benign breast tumors [89,90]. Isla Larrain MT [69] also proved that SM3 resisted abnormally glycosylated MUC1 exerted protection, which might result in a superior prognostic outcome in certain BRCA cases.

- PR81

PR81, also one of the anti-MUC1 mAbs generated through mouse immunization using homogenized BRCA to resist human MUC1, belongs to the mouse IgG1 class which possesses a light kappa chain. PR81 is capable of recognizing and binding to MUC1-expressing BRCA cell surfaces [91]. The PR81 mAb has a stronger binding affinity for the PD (T/S/G) RP sequence of MUC1 TPs [92]. Furthermore, Kouchakzadeh et al. [70] developed 5-fluorouracil (5-FU)-loaded, PR81-labeled bovine serum albumin (BSA) nanoparticles (NPs). According to their results, the NP-labeled PR81 mAb was highly immunoreactive to native oncologic MUC1 or the MUC1-associated peptide, but it almost did not cross-react with non-specific proteins. This composite nanoparticle can induce more BRCA cell death in vitro. In another study, the internalization and growth inhibition rates of the 131I-conjugated PR81 were 60% and 80%, respectively, within MCF7 cells; as a result, it might serve as the candidate treatment for BRCA. Apart from that, PB81 also has a critical effect on BRCA radioimmunoscintigraphy [93,94].

- MIN-C2 and MIN-E6

MIN-C2 and MIN-E6, two mouse IgG antibodies, are capable of recognizing a 45-AA membrane-proximal extracellular region in MUC1 (PSMGFR) on MUC1-expressing tumor cell surfaces, rather than on human stem cell surfaces. It has also been shown that the scFv format of MIN-C2 inhibits MUC1-positive breast ZR-75-1 cell development [95].

- DMB-5F3

DMB chimeric antibodies can combine with MUC1’s SEA domain which contains diverse members, including DMB4F4 Igγ1, DMB4B4 Igγ1, DMB10F10 Igγ1, DMB7F3 Igγ1, DMB5F3 Igγ1, DMB13D11 IgA, and DMB10B7 IgA. Notably, DMB-5F3 has been suggested to have the greatest affinity for the SEA domain and can direct against the α/β junction. It also reacts with BRCA cells and can be internalized in DA3 cells. It exerts specific cytotoxicity against cancer cells, but its activities have not been assessed in vivo, representing a future research direction [73]. Furthermore, humanized chimeric DMB-5F3 conjugated with ZZ-PE38 (ZZ IgG-binding protein after fusion with the Pseudomonas exotoxin) can substantially promote MUC1+ cancer cell cytotoxicity in vitro [72].

- DMC209

As a mouse IgM antibody, DMC209 shows specific binding to MUC1 α/β junction in the presence of these two subunits as interactive molecules within MUC1/TM and MUC1/X proteins in BRCA cells. After SCID mice bearing xenografts of human BRCA MDA-MB-231 cells were injected with the DMC209 mAb, significant tumor suppression was observed in vivo [96]. It has been reported that DMC209 was able to interact with the SEA α/β junction but to a decreased degree of affinity relative to the new antibodies against the MUC1 α/β junction [73,97].

- SKM1

As a group of new humanized scFv, SKM1 was prepared by adopting phage display technology against the MUC1-C recombinant protein. Typically, the SKM1-02/-13/-20 antibodies suppress TNBC cell invasion to a great extent. Of these, SKM1-02 can bind onto the MIN-C2 antibody and BRCA cell surface, and it suppresses MUC1-positive BRCA cell invasiveness while reducing their cell viability [74].

- GGSK-1/30

As an anti-MUC1 IgG1, GGSK-1/30 shows high specificity for TNBC cells, but it does not combine with healthy MUC1 on breast epithelial cells [98]. Stergiou et al. [75] confirmed that its desferrioxamine (Df) conjugated with ^89^Zr produces a diagnostic imaging tool [^89^Zr]Zr-Df-GGSK-1/30, for the early diagnosis of human tumor-associated MUC1 expressing BRCA, which may be used for radioimmunotherapy in future studies.

- AR20.5

As another mouse anti-MUC1 IgG1, AR20.5 (BrevaRex) is produced based on MUC1’s tumor-associated antigen in the backbone of the TP protein. It has a strong reaction with the MUC1 cell surface epitope or its soluble form in diverse tumor cell types [99]. AR20.5 was generated through the immunization of three diver sources, including the culture medium of MCF-7 cells, human fluids, and OC case-derived MUC1. Moreover, it also interacts with 6 AAs in the DTRPAP VNTR region. However, adding one individual GalNAc promotes its affinity for the MUC1 epitope, suggesting that AR20.5 binds to the carbohydrate residue that is involved in recognizing antigens [100]. The AR20.5-MUC1 complex can be formed with MUC1 from MUC1-positive cancer cells or in circulation. In addition, AR2.5 internalization is achieved via dendritic cells (DCs), thus facilitating efficient MUC1 antigen processing, cross-presentation to T-cells, and the later activation of cytotoxic T-cells for eradicating cancer cells [99]. Apart from that, a Phase I trial adopted AR20.5 to induce MUC1-specific immunity for treating advanced adenocarcinoma cases. These cases showed a favorable tolerance, to all doses investigated, with low toxicity [81]. Furthermore, Mehla et al. [101] assessed whether AR20.5 immunization with anti-PD-L1 and Poly ICLC was effective on pancreatic cancer patients. As a result, AR20.5 attained effectiveness as well as long-term anticancer cellular immunity in hosts bearing pancreatic cancer; this effect was even stronger when an ICI and vaccine adjuvant were utilized. The US FDA has approved the use of anti-PD-L1 ICIs to treat BRCA. It can be inferred that the above combination might also affect breast cancer, but this is not yet supported by any research data.

- 3D1

As an anti-oncogenic MUC1-C subunit antibody, 3D1 shows an affinity for MUC1-C ECD (MUC1-C/ED) in the α3 helix (VHDVETQFNQ). Panchamoorthy et al. [71] reported that monomethyl auristatin E (MMAE)-conjugated 3D1 exhibited an efficacy as potent as ADC in eradicating MUC1-positive TNBC cells, with no cytotoxicity in the healthy cells. In addition, in mouse models in vivo and in vitro, 3D1 maintains its specific affinity for MUC1-C/ED as well as its anti-cancer effect. Given the non-toxicity and the above results, humAb 3D1-MMAE ADCs may be developed as a treatment for MUC1-C-overexpressing tumors.

- KL-6

KL-6, a mouse IgG1 mAb that recognizes a sialylated sugar of Krebs von den Lungen-6 (KL-6), is classified as a MUC1-derived glycoprotein antigen and can react with the PDTRPAP sequence, which is the minimal antigenic epitope. The mAb against KL-6/MUC1 can promote MUC1 glycoprotein aggregation in either cell pole, also called a capping formation, giving rise to MUC1-positive BRCA cell death. In addition, anti-KL-6 mAb-induced MUC1 capping is a novel method of developing mAb-based immunotherapies for treating MUC1-overexpressing BRCA. Moreover, the mAb against KL-6 promoted the killer cell-induced cytotoxicity activated by lymphokine, thus playing an auxiliary role in treatment [76,102,103,104].

- VU-2-G7

VU-2-G7 is a type of IgG1 kappa murine mAb developed based on the synthesized 60-mer MUC1 triple TP peptide that contains the GalNAc O linked to threonine within the PDTR region, whereas VU-2-G7’s predominant epitope is present within MUC1 TP’s glycosylated PDTR motif, which shows high abundance in the cancer cell surface of OC/BRCA tissues. Moreover, it was found that VU-2-G7 is effective at recognizing MUC1 overexpression in OC/BRCA tissues and cells to conduct passive immunotherapy [77].

- 5E5

5E5 is a murine IgG1k mAb produced through wild-type (WT) Balb/c mouse immunization using the KLH-conjugated GalNAc-glycosylated MUC1 glycopeptide (VTSAPDTRPAPGSTAPPAHG) [105,106]. The 5E5 mAb was featured with high specificity to BRCA cells and tissues. The above mAbs interact with sTn- or Tn-carrying MUC1 within TPs’ GSTA sequence, but they are unable to identify the T antigen-carrying GSTA epitope [3,107]. mAb 5E5-based immunolabelling suggested that the Tn-MUC1 epitope was expressed within BRCA cells and tissues; meanwhile, mAb 5E5 caused ADCC within T47D and MCF7 BRCA cells [78]. Gong et al. [108] described a new, completely humanized mouse 5E5-antibody-based antibody that specifically targeted the MUC1-Tn/STn epitope. The authors verified the specific recognition of tumor-associated MUC1 epitopes, human NK cell activation, and NK cell-mediated ADCC enhancement in vitro via the above antibodies, thus having the potential to be a therapeutic candidate for BRCA cellular immunotherapy.

#### 3.1.2. Bispecific Antibodies against MUC1

Bispecific antibodies (bsAbs) can simultaneously recognize two distinct cancer epitopes and enhance tumor cell engagement by immune cell components. Thus, theoretically, they may have an improved treatment effect. In recent research, MUC1 is suggested to be the candidate bsAb target in BRCA immunotherapy, and there are three bsAbs that have made preliminary attempts in this regard.

- Anti-MUC1 × Anti-CD3 bispecific antibodies

Many bsAbs have been developed, and they show a high affinity for CD3 and MUC1 antigens located in T-lymphocyte cells. Katayose et al. [82] prepared two bsAbs, namely, MUC1 × CD28 bsAb synthesized using 15E8 (anti-CD28) and MUSE11, together with MUC1 × CD3 bsAb synthesized using OKT-3 (anti-CD3) and MUSE11 (tumor antigen against MUC1). They both reacted well with both MUC1-positive target bile duct carcinoma (BDC) cells and enhanced CD3-positive T-LAK cells’ anticancer effect to a great extent. Apart from that, cancer growth was inhibited within immunodeficient mice bearing BDC xenografts, and a new PD-1 inhibitor conjugated to anti-CD3 and anti-MUC1 bsAbs was analyzed in a Phase II RCT (NCT03524261) in advanced BRCA cases, but no further information is available [83]. The MUC1+CD3 bsAb was synthesized in another work by adopting the variable anti-CD3 antibody TR66 region and anti-TA-MUC1 PankoMab to target MUC1-expressing MCF-7 BRCA cells. As suggested by co-culture analysis, MUC1+CD3 BsAb efficiently enhances the potent anticancer T-cell response while improving cytotoxicity [109].

- Anti-MUC1 × Anti-Ga chelate bispecific antibody

Schuhmacher et al. [84,85] prepared the bs-MAb from the anti-Ga chelate MAb’s F(ab’) fragment and the anti-MUC1 MAb 12H12’s F(ab’) (2) fragments (reacted with most human BRCAs) linked via a bifunctional chemical linker. Additionally, BRCA-bearing nude mice were adopted to test the affinity together with the biokinetics of this as-constructed bs-MAb. This bs-MAb showed a low affinity for BRCA cells, while the binding capacity of cells was high, contributing to the successful improvement of immunoscintigraphic cancer localization within BRCA-, pancreatic cancer-, and colorectal cancer (CRC)-bearing mice, and provided an experimental basis for BRCA radioimmunotherapy.

- Anti-MUC1 × Anti-erbB-2 bispecific antibody

MUC-1, a kind of human epithelial mucin, shows extensive expression within adenocarcinomas (80% of BRCAs are included), whereas erbB-2 overexpression is detected in around 30% of BRCAs. erbB-2 and MUC-1 levels can be partly overlapping but in an uncoordinated manner. Consequently, using antibodies against these two antigens concurrently may add to the number of patients that can benefit from immunotherapy. Moreover, Akewanlop et al. [86] adopted monocyte-derived macrophages mediated by mAb DF3- and DF3xH22 (it’s bsAb) to analyze cytolysis and phagocytosis in cultured human BRCA cells. They found that bsAb DF3xH22 functioned to mediate MUC-1-positive target cell phagocytosis effectively.

### 3.2. MUC1 Targeted CAR T-Cell Therapy

CAR T-cell therapy is conducted on the basis of circulating T-lymphocytes that can subsequently be engineered for expressing CARs; as a result, these T-lymphocytes are capable of recognizing and responding to tumor cells without the need for the major histocompatibility complex (MHC). The in vitro proliferating T-lymphocytes can then be reinfused in the patient for driving anticancer immunity [110]. In a nutshell, T-lymphocytes modified by CAR can integrate mAbs’ specificity with T-cells’ killing and homing abilities. To be specific, CAR T-cell therapy has been recognized with numerous advantages relative to additional cell immunotherapies. First of all, CAR T-cells can be produced by the polyclonal T-cells under non-specific activation. Consequently, the difficulties in isolating and amplifying CD4+ and CD8+ T-cells specific to natural tumors can be solved [111,112]. Second, CAR T-cells are capable of recognizing target antigens without the need for MHC, can identify target cells showing a decreased antigen processing or HLA level, and are suggested to be a critical factor for tumor immune invasion [113,114,115]. Third, the active and specific localization of CAR T-cells are available at cancer sites, and these cells can expand and maintain for a long time following in vivo tumor recognition. Consequently, tumor-associated antigens (TAA)-targeting CAR T-cells possibly show increased efficiency compared with mAbs in generating long-term anticancer responses [116]. CAR T-cells can also cross the blood-brain barrier (BBB) [117], which is extremely conducive to the treatment of cancers involving or migrating to the central nervous system (CNS).

Currently, CAR T-cell therapy is becoming a rapidly developing and extensively used antitumor cell immunotherapy branch. At present, it substantially alters the hematological malignancy landscape, occupying over half of the developing or the already-used cell treatments [118]. However, there were still problems associated with the use of CAR T-cells in treating solid tumors, regardless of its successful treatment of hematological cancers [119,120]. Typically, CAR T-cell therapy, when it is used in treating solid tumors, is associated with the primary challenge of identifying suitable tumor target antigens with little or no expression in healthy tissues (especially in vital organs). What is worse, for the specific CAR T-cell type, only the identification of several receptors on target cells for complete activation is required. To avoid off-target effects, as well as the relevant toxicity, it is necessary to select a suitable target antigen (namely, one with overexpression in tumor cells but little or no expression in healthy samples) [121,122,123]. Intratumor heterogeneity on the antigen level is also a problem related to the use of CAR T-cell therapy for treating solid cancers and it may result in tumor escape [123,124]. It should be mentioned that an additional challenge is related to the immunosuppressive TME [125]. 

MUC1 accounts for a significantly related antigen targeting BC, and its expression can be detected in 90% of BRCA cases, especially in PgR- and ER- groups [126]. Interestingly, tMUC1, MUC1 in an abnormally glycosylated tumor form, shows overexpression in over 95% of TNBC cases, but it is rarely expressed in healthy mammary tissues. Likewise, TnMUC1, MUC1 in an abnormally glycosylated tumor form, also shows a high expression level in TNBC. Notably, CAR T-cells that can recognize this glycoform can be recognized as real target CAR T-cells for TNBC [127,128]. Collectively, MUC1 (tMUC1and TnMUC1) is a critical target that can be used to treat BRCA by CAR T-cell therapy, especially for TNBC with a highly specific tumor antigen.

In 2008, Wilkie et al. [129] at King’s College London School of Medicine constructed a MUC1 CAR containing the CD28/OX40/CD3zeta endo domain by adopting the HMFG2 mAb-derived scFv. As a result, CAR T-cells generated proinflammatory factors for eradicating the MUC1(+) cancer cells and grew after being stimulated by MUC1. CAR T-cell therapy helps to significantly postpone cancer growth in vivo. Their research was the first to demonstrate that CAR-grafted T-cells could be used to target the almost-omnipresent MUC1 tumor antigen. However, HMFG2-based CAR interacts with MUC1-expressing healthy epithelium as well.

In 2016, Posey et al. [128], from the University of Pennsylvania, prepared a high-affinity antibody (5E5) by the co-stimulation of 4-1BB with CAR, which was later directed to MUC1’s specifically truncated O-glycopeptide epitopes whose expression was not detected in normal tissues. Neither the CAR nor the 5E5 antibody binds to the non-glycosylated MUC1 protein, in contrast to HMFG2. Furthermore, they do not combine with glycosylated MUC1 normally; instead, they show specific binding to MUC1’s Tn glycoform (TnMUC1) by recognizing GSTAP, the unique short peptide sequence motif. In addition, 5E5 CAR T-cells are not cytotoxic to normal primary cells; as a result, they can discriminate the expression of the TnMUC1 glycoform in the tumor from that in normal cells. Additionally, one clinical trial (NCT04025216) is also being conducted to examine the role of CAR T-cells in targeting diverse TnMUC1-expressing tumor types.

In 2018, Bajgain et al. [130] at Baylor College of Medicine constructed an inverted cytokine receptor linking MUC1-specific CARs by using the HMFG2 scFv. At tumor sites, transgenic T-cells exhibit selective expansion in the presence of co-stimulatory signals, thus producing strong and persistent in vivo and in vitro cancer control. The authors indicated that it was feasible to use transgenic T-cells to target BRCA so as to suppress the TME.

In 2019, Zhou et al. [127] at the University of North Carolina at Charlotte prepared the new CD28-costimulated MUC1 CAR-based mAb TAB004. The xenograft model and human TNBC cells were infused with MUC28z CAR T-cells. These CAR T-cells were able to effectively lyse TNBC cells depending on tMUC1 in vitro while efficiently reducing TNBC development in vivo. Notably, mAb TAB004 can identify the changed glycosylated epitope in the MUC1 TP sequence, with the STAPPVHNV sequence being the binding epitope, suggesting the high specificity of TAB004 for tumor-associated MUC1 and its inability to recognize healthy epithelium. Therefore, MUC28z CAR T-cells based on TAB004 have the great potential to treat TNBC expressing tMUC1 and highly specific tumor antigens, with minimal injury to the healthy mammary epithelium.

In 2021, Zhai et al. [131] at Soochow University replaced traditional αβ T-cells with Vγ9Vδ2 T-cells that are easier to obtain and expand and analyzed the antigen-specific anticancer effect of MUC1-Tn antigen-targeting CAR-Vγ9Vδ2 T-cells. As suggested by cytotoxicity tests on BRCA cells, CAR-Vγ9Vδ2 T-cells had potent effects on cancer cell lysis upon specific antigen, and such effects might be comparable to or more potent than CAR-αβ T-cells. Consequently, according to the study, Vγ9Vδ2 T-cells modified by MUC1-Tn CARs could be candidates for new forms of anti-BRCA allogeneic immunotherapy. In the same year, Nalawade et al. [132] at Baylor College of Medicine published similar research results. To overcome the problem of the persistently suppressed CAR T-cell activity by myeloid-derived suppressor cells (MDSCs) within the TME of BRCA, the authors prepared a MUC1-targeting CAR T-cell using a new chimeric costimulatory receptor targeting the TNF-associated apoptosis-inducing ligand receptor 2 (TR2) distributed in MDSCs. According to their findings, the TR2 receptor co-expressing CAR T-cells showed an increased ability to resist BRCA that contained tumor-promoting and immunosuppressive MDSCs, leading to TME remodeling while promoting T-cell growth at the tumor site, thus showing clinical translation potential.

To enhance the efficacy of MUC1targeted CAR T-cell therapy for BRCA, in addition to designing unique CAR structures to improve targeting, multiple antigens targeting CAR T-cells can also be employed, such as dual CAR T-cells [133,134], unique T-cells specifically co-expressing two individual CARs in two distinct TAAs under genetic manipulation, thus enhancing cancer cell targeting accuracy while enhancing the anticancer effect of CAR T-cells. Meanwhile, dual targeting is an attractive method of CAR T-cell therapy because of its effect on reducing treatment-induced toxicity while promoting intra-tumor T-cell survival. Moreover, decreased toxicity may be related to the optimal tumor specificity and T-cell homing, whereas mouse models of BRCA show that CAR T-cells target both transmembrane glycoprotein mucin 1 (MUC1) and human epidermal growth factor receptor 2 (HER2). Furthermore, Wilkie et al. [135] examined the cytotoxicity induced by the co-expression of MUC1- and ErbB2-specific CARs. According to their results, the dual targeting of CARs promoted the growth of CAR T-cells while effectively killing tumor cells co-expressing MUC1 and ErbB2.

At present, clinical trials are being conducted to investigate the anti-BRCA effect of MUC1–CAR T-cell therapy. Minerva Biotechnologies Corporation began to recruit metastatic BRCA patients in a Phase I trial (NCT04020575) in 2019 to investigate the MTD and the safety of autologous CAR T-cells that targeted the MUC1 antigen in the cleaved form. In addition, Tmunity Therapeutics conducted a Phase I, open-label, multicenter RCT (NCT04025216) to investigate TnMUC1 CAR T-cells’ feasibility, safety, and preliminary effects and tolerability among TNBC cases.

### 3.3. Vaccine-Based Therapies

Tumor vaccine therapy is a kind of active tumor immunotherapy. It utilizes a specific antigen to promote the antigen-specific immunoreaction inside the human body and activates the immune system in the patient for eradicating cancer cells [136]. As early as 2010, the FDA approved the first autologous active immunotherapy drug and the first truly therapeutic cancer vaccine Provenge/Sipuleucel-T to treat advanced prostate cancer (PCa) [137]. This marked the entry of tumor treatment into the era of vaccine treatment, which was followed by remarkable results in tumor vaccine therapy in the field of BRCA treatment [136,138]. In December 2020, the SABCS conference announced a prospective, multicenter, single-blind, placebo-controlled, Phase IIb RCT on the new BRCA vaccine GP2, i.e., HER2 targeted therapy plus GP2 vaccine treatment, resulting in a five-year disease-free survival rate of 100% and a recurrence rate of 0% in patients with BRCA (NCT03014076), which means that all patients who received the vaccine treatment regimen experienced a clinical cure. Partially, vaccine therapy, similar to a beacon, guides researchers seeking to win the battle against BRCA, and MUC1 is one of their weapons. Generally, tumor vaccines can be classified as subunit, DC, nucleic acid, or viral vector vaccines [139]. Here, the recent advances in different types of BRCA vaccines based on MUC1 are described.

#### 3.3.1. Subunit Vaccines

Subunit vaccines have critical effects on suppressing cancer migration and development. After exposure, the APDTRP epitope [140] with abnormally glycosylated MUC1 is identified via several antibodies against MUC1, thus activating cytotoxic T-lymphocytes (CTLs) specific to tumor antigens. Consequently, synthesized MUC1 peptides are utilized as subunit vaccines to induce adaptive immunity. Subunit vaccines contain tumor-related human MUC1 glycopeptides inducing potent anticancer humoral immunity in mice. However, MUC1 glycopeptides have low immunogenicity, which, together with the immunosuppressive microenvironment and the immune tolerance of the body, restricts their application as therapeutic vaccines for humans. Recently, researchers have focused their MUC1 BRCA vaccine research on adjuvant subunit vaccines, agonist-coupled subunit vaccines, and combination vaccines. A research team from Johannes Gutenberg-University Mainz has carried out continuous and in-depth research in this field. They designed a vaccine composed of MUC1 TP peptide-bound Tn and sTn antigens combined with the tetanus toxoid. This vaccine triggered potent immunity in huMUC1-transgenic and WT mice, with no auto-aggressive adverse reactions. Each anti-serum displayed a nearly equal binding affinity for human BRCA cells, with comparable elevations in the amounts of activated B-cells, CD4+ T-cells, and DCs within lymph nodes [141]. In addition, according to their further research, the anticancer-associated human MUC1 preventive vaccination specifically induced humoral immunity postponed cancer development, and boosted the BRCA-bearing mouse survival [142]. Moreover, their group, for the sake of improving antigen presentation and consumption by targeting DCs and macrophages with a positive mannose-receptor, also developed a MUC1 antitumor vaccine bound to the covalently linked divalent mannose ligands, thus inducing substantially enhanced specific IgG immunity in mice compared with a non-mannosylated control vaccine. The introduction of mannose also elevated the amounts of CD4+ T-cells, DCs, and macrophages within local lymph organs [143]. As a result, a MUC1 vaccine equipped with covalently linked divalent mannose ligands and a tumor-related huMUC1 vaccine combined with the tetanus toxoid are promising anti-BRCA vaccines. Beyond that, the tumor angiogenesis inhibitor mVEGF165b monoclonal antibody [144] and the COX pathway inhibitor indomethacin [145] may also be ideal immunization adjuncts for enhancing the immune efficacy of MUC1-based BRCA subunit vaccines.

However, other researchers believe that MUC1 peptide-based adjuvant subunit vaccines are not effective enough for clinical use. Thus, they are trying to find more effective agonists to enhance the BRCA therapeutic effect of the MUC1 vaccine. In this regard, there are several studies worthy of attention. Liu et al. [146] synthesized a new MUC1 peptide vaccine conjugated with the toll-like receptor 7 (TLR7) agonist (denoted as T7-MUC1), which induced potent immunity and anticancer activity in a mouse model of BRCA. Moreover, T7-MUC1 remarkably promoted cytokine production in vitro within mouse splenic lymphocytes and bone marrow-derived DCs, while inducing a DC-cytokine-mediated anticancer killer response in MUC1-overexpressing cells. Further, T7-MUC1 conjugate-immunized mice decreased 4T1 tumor weights by ≥70% relative to the control. In their opinion, the T7-MUC1 vaccine suppressed cancer development within mice and might serve as a candidate anti-BRCA immunotherapeutic strategy. Glaffig et al. [147] at Johannes Gutenberg-University Mainz established a completely synthesized MUC1 glycopeptide anticancer vaccine conjugated to poly (inosinic acid:cytidylic acid), poly(I:C), which was used as a structurally defined adjuvant to activate TLR3. The as-constructed vaccine triggered outstanding IgG antibody titers and potently combined with BRCA cells that expressed tumor-related MUC1. Apart from humoral immunity, this poly(I:C) glycopeptide vaccine also resulted in a pro-inflammatory environment while eliciting potent cellular immunity for the eradication of cancer cells. Based on the above immunological analysis, poly(I:C) substantially promotes anti-MUC1 anticancer vaccine immunity as a synthesized adjuvant (NCT00986609).

#### 3.3.2. Dendritic Cell (DC) Vaccines

DCs are immune checkpoints (ICPs) that have the essential effect of triggering and modulating antigen-specific immunity. In the TME, as professional antigen-presenting cells (APCs), DCs can process and present TAAs via MHCI/II complexes to activate T-cells. The action mechanism underlying DC vaccines may be associated with stimulating and supporting immunity for the exclusive elimination of cancer cells from the body. The common procedure of producing DC vaccines is the separation of DCs or corresponding precursors in the human blood, subsequently using in vivo autologous tumor antigens and a cytokine cocktail for maturating and activating cells, and finally administering these cells to the body. DCs function to present antigens for CD4+/CD8+T-cells within lymph nodes and then activate the humoral and cellular adaptive immunity. Thereafter, certain cancer cells can be recognized and killed by cytotoxic T-cells [148]. In 1996, an initial RCT on the DC vaccine was initiated. At present, there are over 250 RCTs regarding DC vaccine-based anticancer therapies registered at ClinicalTrials.gov (accessed on 11 May 2022).

The existing DC vaccines can strengthen CD8+ T-cell and effector responses, but they still cannot attain a satisfying effect. Two main problems must be solved before successful DC vaccines can be developed: the limited functions of the frequently adopted monocyte-derived DCs (MoDCs) and the tumor-triggered immunosuppression [149]. For early tumor patients, whose peripheral DCs are more than doubly decreased, the degree of the influence of the tumor immunosuppressive microenvironment induced by DCs dramatically declines relative to normal controls (<0.5% of peripheral blood mononuclear cells, PBMCs); by contrast, for advanced tumor cases, the number of DCs declined by four-times relative to the controls. In addition, the tumor releases the IL-6, IL-10, VEGF, gangliosides, and/or macrophage colony-stimulating factor (M-CSF), thus suppressing DC activity and differentiation both in vivo and in vitro. Consequently, it is impossible for tumor cases to eradicate tumors through DC activation and differentiation in vivo [150]. 

MUC1 that shows abnormal expression in cancer cells might be a strong chemoattractant for immature DCs. A study has shown that DCs can bind to and internalize MUC1’s short sialylated carbohydrates, which activated and maturated DCs. However, MUC1-induced DCs did not trigger the type-1 response necessary for tumor rejection [151]. As suggested in one work, a tumor-bearing MUC1 damaged DC activity and differentiation by upregulating CD1a/CD206, two factors related to the immature phenotype of DCs. Additionally, when the tumor-bearing MUC1 existed, DCs produced excessive IL10 rather than IL12, even after being stimulated by LPS. This changed the balanced IL12/IL10 secretion, thus impairing APC’s ability to produce autologous and allogeneic immune reactions [151]. Therefore, a tumor that bears MUC1 may develop immune escape by damaging DC maturation as well as differentiation.

MUC1-based DC vaccines can be prepared through the fusion of DCs with cancer cells that express MUC1, transfection using MUC1 RNA, and pulsing using the MUC1 TP peptide [152]. Initially, researchers explored the feasibility of MUC1-targeted DC vaccines in BRCA treatment. In 2002, Pecher et al. [153] at the Free University of Berlin and Universitätsklinikum Benjamin Franklin, reported the Phase I/II clinical trial results of MUC1-transfected DCs as a vaccine. The MUC1 cDNA-transfected human autologous DC vaccines were adopted to treat ten advanced BRCA, papillary, or pancreatic cancer cases. Among them, three showed delayed-type hypersensitivity (DTH) specific to the vaccine. Afterward, four cases exhibited increased CD8+ T-cells (which secreted mucin-specific IFN-gamma) by 2–10 times. They also showed that a vaccine that comprised DCs transfected with an autologous gene was feasible and safe and that it induced immune responses in advanced cases or in those who did not receive treatment. In 2003, Kontani et al. [154] at Shiga University of Medical Science analyzed the immunotherapeutic efficacy of a MUC1 tumor antigen-targeting DC vaccine. In addition, the clinical trial revealed that this prepared vaccine markedly extended MUC1-expressing patient survival (either BRCA or LC patients) compared with MUC1-negative cases (16.75 versus 3.30 months). Additionally, tumor marker expression decreased, tumor size was reduced, and malignant pleural effusion disappeared in many MUC1-expressing cases (7/9), indicating the sufficient immunogenicity of MUC1 in eliciting potent anticancer immunity and the usefulness of MUC1-targeting DC vaccines in anticancer immunotherapy.

Recently, researchers have turned their attention to the study of compound vaccines with cofactors to improve the therapeutic effect of the vaccine. Qin et al. [155] found the suppressor cells with cytokine signaling 1 (SOCS1)-knockdown plus MUC1-CRT-overexpression promoted cytokine generation as well as T-cell growth via DCs, thus enhancing immune response. That work sheds more light on the development of more potent MUC1-based DC vaccines against BRCA.

#### 3.3.3. Nucleic Acid Vaccines

Nucleic acid cancer vaccines are fascinating because they are safe, stable, and inexpensive. Broadly, these vaccines contain RNA- or DNA-encoded antigens and are thus further classified as RNA or DNA vaccines, which adopt diverse mechanisms to deliver therapeutic agents. 

A DNA vaccine contains plasmids that include a potent promoter, an intron, an appropriate terminator, and several gene insertion cloning sites. In the host, the transcription and translation of the DNA plasmid are performed, so an encoded antigen, usually the protein tumor marker that is processed into peptides, can be generated, and finally presented onto the surface of antigen-presenting cells (APCs) in hosts with MHC molecules. Afterward, neoantigen-specific T-cells can show the specific recognition of the peptide–MHC complex, thereby inducing cellular immunity in the host to resist specific antigen-bearing cancer cells [156]. Furthermore, these vaccines are lowly immunogenic, can be repeatedly injected, and induce extended antigenicity with immunity amplification, relative to additional vaccine types [157,158].

MUC1-targeting DNA vaccines are an extensively investigated immunotherapeutic approach in mice. The MUC1-encoding recombinant eukaryotic expression vector will be intramuscularly injected directly; as a result, the MUC1 protein is substantially and specifically expressed, thus specifically inducing cellular and humoral immunity within the animal model [159].

As reported, applying MUC1 DNA vaccination only cannot sufficiently overcome cancer challenges. Researchers are searching for methods to improve DNA vaccines, such as adjuvant protein fusion during preparation and the use of immune modulators to enhance immunogenicity. For instance, adding IL-18 triggered the activation of NK cells and effectively controlled a tumor [160]. In addition, combining a MUC1 vaccine with mANT2shRNA-1 (short-hairpin RNA) therapy, compared with a single therapy, induced highly potent anti-MUC1 immunity and anticancer activity to resist benign MUC1-positive mammary fibroadenomatosis cell (murine melanoma cell) tumors [161].

Under the COVID-19 pandemic background, the development of an mRNA-based vaccine is a popular area of research, and mRNA-based cancer vaccines have attracted more attention. mRNA was first only selected as a TAA-encoding template in anticancer immunotherapy; however, it is versatile and variable in design and is now recognized to be of unlimited therapeutic potential. mRNAs are now utilized for (1) delivering tumor-specific mAbs to block ICIs, as well as the corresponding fragments to produce bs-Abs and chimeric antigen receptors, (2) delivering toxic proteins that induce tumor cell mortality, (3) modulating tumor-related DCs, (4) modulating the immunosuppressive TME, (5) modulating cytokines within the TME, and (6) generating tumor T-cells [162]. One advantage of mRNA-based vaccines lies in the capability of inducing cellular and humoral immune responses, especially by inducing CD8+ T-cell responses which are important for resisting tumors [163]. Meanwhile, different from DNA vaccines, mRNA vaccines will not induce severe adverse reactions, e.g., integrating into the patient’s genome, possibly including insertional mutagenesis, gene disruption, carcinogenesis, and cell death. Additionally, mRNAs exert their activities within the cytoplasm, without penetrating target cell nuclei to facilitate delivery. Finally, mRNA vaccines can be rapidly and massively produced, and the desired products are highly yielded under in vitro conditions, which is their most attractive advantage. mRNA vaccines have also been simplified, thus substantially reducing complications related to biological vaccine generation, such as environmental risk, genetic variability, and infectious agent processing [156].

In 2018, Liu et al. [164] from the University of North Carolina at Chapel Hill prepared NPs for delivering a tumor antigen MUC1-encoding mRNA vaccine into DCs within lymph nodes, which thereby activated and expanded tumor-specific T-cells. The anticancer activity was further enhanced by combining anti-CTLA-4 (cytotoxic T-lymphocyte-associated protein 4) mAb with the mRNA vaccine. Moreover, as suggested by in vivo research, the mannose receptor-targeting NP-based mRNA vaccines expressed tumor antigens within lymph node-derived DCs, suggesting that the NP vaccine induces a potent antigen-specific T-lymphocyte response with in vivo cytotoxicity for the resistance of TNBC 4T1 cells. Combining vaccine-based immunotherapy with anti-CTLA-4 mAb might dramatically promote anticancer immunity in comparison with either treatment alone. 

Collectively, Table 2 lists the MUC1-based BRCA vaccines reported in the aforementioned RCTs.

## 4. Conclusions and Future Perspectives

MUC1 has been studied in the diagnosis and treatment of BRCA for more than 40 years and in the immunotherapy of BRCA for almost 20 years. In the last five years, especially, with the rise of CAT-T therapies and cancer vaccine therapies, MUC1-based immunotherapy has gained renewed attention and has been studied all over the world [3]. Although many encouraging research results have been achieved, there are still challenges to be overcome before MUC1 immunotherapy can be used in the clinical treatment of BRCA.

The MUC1 target itself has been surveyed. MUC1 was initially recognized to be the almost perfect anticancer immunotherapeutic target, due to its advantages that allow the specific anticancer immune response to be induced within BRCA. However, is MUC1 an ideal target for BRCA immunotherapy? Firstly, there are diverse isoforms of MUC1 that are associated with breast, prostate, and ovarian cancer. Given these isoforms, it is infeasible to develop treatments depending on the immunogenic epitope-targeting agent within TPs. Secondly, even though the abnormally glycosylated MUC1 can be frequently observed in tumors, it may not exhibit the universal glycosylation pattern in different cancers. Particularly, T47D BRCA cells can express high glycosylated MUC1 levels with glycoside residue-masked immunodominant tumor-related epitopes [165]. In different oncological diseases, the availability of varying glycosylation patterns may induce markedly reduced activity as well as the efficacy of universal MUC1-based immune agents. In addition, is the targeting of muc1 reliable? As a heterodimeric protein, MUC1-N in MUC1, which carries an antigenic determinant needed for immunotherapy, will non-covalently bind to its MUC1-C [49,166]. Under certain conditions, MUC1-N will be dissociated from the cell surface and lead to the off-target and non-target effects of MUC1-targeted immune drugs. Therefore, in the future, exploring the specific and stable MUC1 isoforms of BRCA should be carried out.

After finding better BRCA-related MUC1 targets, a second problem to be solved by investigators of MUC1-based immunotherapy is to search for a way to naturally or artificially generate T-cells with receptors that can specifically recognize BRCA-related MUC1. Here, it should be noticed that two major directions must be adopted in relevant studies in this field. Firstly, approaches to the natural generation of T-lymphocytes must be found. For example, producing antigen-presenting cells with high immunogenicity can stimulate T-lymphocytes to recognize and eliminate cancer cells that carry the antigen presented. Secondly, T-lymphocytes may be artificially produced with no antigen presentation; in other words, T-cell-specific chimeric receptors highly specific to tumor-related MUC1 must first be created.

Another major problem of MUC1 targeting BRCA immunotherapy is immunosuppression. As for MUC1-based immunotherapy, mucin is involved in developing immunosuppression (the recognizing agent can bind to MUC1), which thus suppresses immune response development [167]. Additionally, MUC1 represents the critical immunosuppression modulator at the cell and whole-organism levels [168]. Considering that, adding immunostimulatory factors or immunosuppressive blocking factors to MUC1 immunotherapy drugs is effective in terms of enhancing the effect of immunotherapy. Moreover, the MUC1-mediated tumorigenic signaling, tumor rejection, and immune invasion mechanisms need to be illustrated and counteracted. Nevertheless, the best clinical outcome will be obtained through integrating immunotherapy with radiotherapy/chemotherapy/hormonal therapy, targeting the signaling of MUC1, double-targeting TAAs for enhancing specificity, and utilizing the more potent/specific adjuvants.

In short, the existing information shows that MUC1 is one of the most important targets for BRCA immunotherapy, and further investigations on MUC1 will likely provide a key to opening a “door of hope” in anti-BRCA immunotherapy.

## Figures and Tables

**Table 1:** Anti-MUC1 antibodies and antibody-based clinical trials (in BRCA).

Antibody	Epitope	Immunogen	Clinical Trial Number	Study Phase	Ref.
huHMFG1 (AS1402)	Epitope (PDTR) within the VNTR region	Human milk fat globule membrane	NCT00096057	Ⅰ	[57]
			NCT00770354	Ⅱ	[58]
TAB004	STAPPVHNV within the TR sequence	Protein lysate from MUC1-expressing tumors that developed in MUC1 transgenic mice (PDA mice) that expressed human MUC1			[59,60,61,62]
BrE-3	Amino acid sequence TRP on the tandem repeat and with a low carbohydrate	HMFG	NCT00007891	Ⅰ	[63,64,65,66,67]
DS6	O-linked glycans with 2,3-sialylated and 1,4-galactosylated termini in VNTR	Human serous ovarian carcinoma	NCT01156870	Ⅰ	[68]
			NCT02984683	Ⅱ	
SM3	Unglycosylated PDTR motif	Mucin molecule purified from human skimmed milk			[69]
PR81	MUC1 tandem repeats at the PD(T/S/G) RP sequence	Homogenized breast cancerous tissues			[70]
MIN-C2 and MIN-E6	A 45 amino acid membrane-proximal extracellular region of MUC1 from amino acid 1110 to 1155 (PSMGFR)	Synthetic peptideGTINVHDVETQFNQYKTEAASRYNLTISDVSVSDVPFPFSAQ SGA			
3D1	MUC1-C/ED at the α3 helix (α3: VHDVETQFNQ)	MUC1-C/ED protein with complete Freund’s adjuvant and CpG adjuvant			[71]
DMB-5F3	SEA domain	Recombinant bacteria extracellular domain of MUC1-X protein with incomplete Freund’s adjuvant			[72]
DMC209	α/β junction SEA	Injected DNAs consisted of either the pCL-MUC1/TM or pCLMUC1/X expression vector plasmids			[73]
SKM1	MUC1-C	Recombinant human MUC1-C protein containing 45 and 58 amino acids			[74]
GGSK-1/30	hu(TA)MUC1-glycopeptide pattern	22mer huMUC1 peptide sequence of the VNTR region glycosylated with STN on serine-17 tetanus toxoid			[75]
KL-6	A sialylated sugar of Krebs von den Lugen-6 (KL-6) PDTRPAP sequence	Human lung adenocarcinoma			[76]
VU-2G7	PDTR motif	Synthetic 60-mer MUC1 triple tandem repeat peptide with N-Acetylgalactosamine (GalNAc) O-linked to the threonine			[77]
5E5	Tn or STn in the tandem repeat domain	GalNAc-glycosylated MUC1 glycopeptide (VTSAPDTRPAPGSTAPPAHG) conjugated to KLH			[78]
BW835	TF linked to threonine within the VTSA-peptide	MCF-7 and SW-613 breast carcinoma cell lines			[79,80]
mAb-AR20.5	DTRPAP and DTnRPAP	MUC1 from an ovarian cancer patient, derived from human fluids and MCF-7 cell culture medium		Ⅰ	[81]
Anti-MUC1 × CD3 (MUSE11 × OKT-3)	Epitope (PDTR) within the VNTR region	Circulating pancreatic cancer-associated antigen	NCT03524261	Ⅱ	[82,83]
(MUSE11 × OKT-3)					
(MUSE11 × OKT-3)					
(MUSE11 × OKT-3)					
Anti-MUC1 × Ga	The F(ab’) (2) fragments of the anti-MUC1 MAb 12H12	A mouse IgG1 raised against tumor cells from a human mammary carcinoma			[84,85]
Anti-MUC1 × erbB-2	MUC-1 tandem repeat	MAb DF3-P was generated against the tandem repeat of the DF3 protein (VTSAPDTRPAPGSTAPPAHG)			[86]

**Table 2:** MUC1-based cancer vaccine clinical trials (in BRCA).

Trial Number	Intervention	Study Phase	Status	Year
NCT03384316	Multi-Targeted Recombinant Ad5 (CEA/MUC1/Brachyury) Based Immunotherapy Vaccine	Phase 1	Completed	2017
NCT02270372	ONT-10 and Varlilumab combination	Phase 1	Completed	2014
NCT01556789	ONT-10	Phase 1	Completed	2012
NCT01978964	ONT-10	Phase 1b	Completed	2013
NCT00986609	poly-ICLC + MUCI peptide vaccine	Early Phase 1	Completed	2009
NCT00640861	MUC-1 peptide vaccine	Early Phase 1	Completed	2008
NCT00706615	Ad-sig-hMUC-1/ecdCD40L vaccine	Phase 1	Withdrawn	2008
NCT00179309	PANVAC-V + PANVAC	Phase 2	Completed	2005
NCT00004156	MUC1-KLH vaccine	Phase 1	Completed	2004
NCT00088413	PANVAC-V + PANVAC	Phase 1/2	Completed	2004
NCT00071942	rV-MUC-1 vaccine	Phase 1	Terminated	2003
NCT00030823	Globo-H-GM2-Lewis-y-MUC1-32(aa)-sTn(c)-TF(c)-Tn(c)-KLH conjugate vaccine	Not Applicable	Completed	2003
NCT00003761	rV-DF3/MUC1	Phase 1	Completed	2003

## Data Availability

Not applicable.
